# Developing patient-centred care: an ethnographic study of patient perceptions and influence on quality improvement

**DOI:** 10.1186/s12913-015-0770-y

**Published:** 2015-04-23

**Authors:** Alicia Renedo, Cicely Marston

**Affiliations:** Department of Social & Environmental Health Research, Faculty of Public Health & Policy, London School of Hygiene and Tropical Medicine, 15-17 Tavistock Place, London, WC1H 9SH UK

**Keywords:** Quality healthcare, Quality improvement, Service improvement, Patient and public involvement, Patient and public engagement, User involvement, Neoliberalism, Participation, Patient-centred health systems

## Abstract

**Background:**

Understanding quality improvement from a patient perspective is important for delivering patient-centred care. Yet the ways patients define quality improvement remains unexplored with patients often excluded from improvement work. We examine how patients construct ideas of ‘quality improvement’ when collaborating with healthcare professionals in improvement work, and how they use these understandings when attempting to improve the quality of their local services.

**Methods:**

We used in-depth interviews with 23 'patient participants' (patients involved in quality improvement work) and observations in several sites in London as part of a four-year ethnographic study of patient and public involvement (PPI) activities run by Collaborations for Leadership in Applied Health Research and Care for Northwest London. We took an iterative, thematic and discursive analytical approach.

**Results:**

When patient participants tried to influence quality improvement or discussed different dimensions of quality improvement their accounts and actions frequently started with talk about improvement as dependent on collective action (e.g. multidisciplinary healthcare professionals and the public), but usually quickly shifted away from that towards a neoliberal discourse emphasising the role of individual patients. Neoliberal ideals about individual responsibility were taken up in their accounts moving them away from the idea of state and healthcare providers being held accountable for upholding patients’ rights to quality care, and towards the idea of citizens needing to work on self-improvement. Participants portrayed themselves as governed by self-discipline and personal effort in their PPI work, and in doing so provided examples of how neoliberal appeals for self-regulation and self-determination also permeated their own identity positions.

**Conclusions:**

When including patient voices in measuring and defining ‘quality’, governments and public health practitioners should be aware of how neoliberal rationalities at the heart of policy and services may discourage consumers from claiming rights to quality care by contributing to public unwillingness to challenge the status quo in service provision. If the democratic potential of patient and public involvement initiatives is to be realised, it will be crucial to help citizens to engage critically with how neoliberal rationalities can undermine their abilities to demand quality care.

## Background

Healthcare organisations increasingly seek to improve quality by focusing care on patient needs and preferences, with ‘patient-centeredness’ recognised as a domain of quality in its own right [1-3]. There is also a growing emphasis on the need to include patient voices in measures of healthcare quality [4]. Yet patients are often excluded from participating in planning and delivering healthcare quality improvements [5]. We examine the case of patient involvement in healthcare quality improvement interventions (i.e. initiatives that use systematic approaches to make changes in service provision to improve patient outcomes and experience [6]). We focus on how patients construct ideas of ‘quality improvement’ when collaborating with healthcare professionals in improvement work, and how they use these understandings when attempting to improve the quality of their local services. Patient participants involved in quality improvement activities are key social actors positioned at the interface between the public and healthcare professionals, and as such offer an excellent entry point for starting to understand quality improvement from a public rather than solely from a professional perspective. Patient participant perceptions of ‘quality improvement’ can then help illuminate wider ideas about ‘quality’ itself.

Measuring quality of healthcare is a key policy and academic concern [7], yet even while ‘continuous quality improvement’ is widely considered an “ideal” characteristic of healthcare systems ([8]: 53), ‘quality’ is rarely defined at all in the healthcare literature [9,10], and definitions of ‘quality improvement’ are inconsistent [10-13].

There is no consensus around what if any of the existing definitions of quality should be used [9,14], but the concept is generally defined from the perspective of health professionals [15] and focused on effective and efficient function of healthcare systems and providers [7]. Some survey research collates patient views on aspects of care they consider important (e.g. [16]), but with a few notable exceptions (e.g. [17-19]), indicators in these studies are defined a priori by clinical experts without patient involvement [17].

Even given the lack of consensus about what quality of care means [9,14], the quality improvement movement has continued to grow since its emergence nearly four decades ago. Healthcare services are under pressure to deliver quality care and an “industry” [9: 246] of improvement has been developed to help them. An academic quality improvement discipline has emerged [20] and organisations and initiatives have been established to assess quality, to support capacity for quality improvement and to build ‘cultures of improvement’ within healthcare organisations (e.g. The Health Foundation in the UK, The Institute for Healthcare Improvement in the USA, the Canadian Foundation for Healthcare Improvement) [21]. There is usually limited if any public participation in defining and setting priorities for quality improvement [17] and the way patients understand ‘quality improvement’ remains unexplored.

The increasing emphasis on quality of care is part of a wider consumerist movement towards market-orientated neoliberal models of healthcare [22], which emphasise patient choice, individual responsibility, and agency with respect to personal health and wellbeing [23]. The now widely-accepted assumption that patients should have more agency and be more responsible for their own health has given rise to a strong health promotion agenda focused on self-management and ‘expert-patient’ approaches. Securing better health outcomes is now to be achieved not only through improving health systems but also through improving individual citizens themselves, ‘empowering’ them to become responsible ‘expert’ patients. Under this ‘expert patient’ model, neoliberalism operates as “a kind of self-imposed disciplinary code” ([24]: 381) upon patients. Underpinned by an ethos of self-help [25,26] patients are now exhorted to use their own resources (e.g. time and knowledge) and to self-regulate to ensure they remain healthy and avoid unnecessary costs to health systems. This ‘expert-patient’ idea has been criticised for trying to inculcate an idealised model of patient citizenship [27] which narrowly targets individual patients, seeking to improve their psychological dispositions and conditions (i.e. knowledge, attitudes and behaviour) while neglecting the role that context plays in shaping health [28].

By contrast the quality improvement approach moves away from the focus on discrete actions and individual actors, and towards a wider examination of interdependencies and relationships between multiple systems (e.g. provider teams, hospital units, general practice, commissioners and policy context) and processes [29]. In this approach, fostering sustained improvements requires complexity to be addressed [29] and requires attentiveness to the intricate web of interactions between the actors and contexts that shape healthcare [30]. A quality improvement approach is based on the idea of “collaboratives” [12] of professionals and other stakeholders (including patients) working interdependently across intersectoral and interorganisational boundaries to deliver improvement [31].

Against the backdrop of rising emphasis on quality improvement, the need for patient and public involvement in planning and designing care has come to the fore, with involvement in shaping healthcare and access to quality healthcare increasingly framed as rights of citizenship [32,33]. Not only is understanding quality improvement from a patient perspective crucial for successful delivery of patient-centred improvements in care, but the ways patients – including those involved in quality improvement work – define how quality should be achieved remains unexplored. Without understanding how patients construct ideas of how to improve quality of care, policies and improvement interventions risk being disproportionately orientated towards service provider priorities and disconnected from the everyday realities of patients’ lives. Attention to how quality improvement is defined from patients’ perspectives is crucial for citizens to become “makers and shapers” not simply “users and choosers” [34] of quality health services. Failure to take patient views into account risks undermining healthcare systems’ accountability and responsiveness to citizens and their rights to quality healthcare.

In this paper, we examine what ‘quality improvement’ means to patients who participate in shaping healthcare services, and how they use these understandings when attempting to improve quality of care. We draw conclusions about the ideas of ‘quality’ underpinning the patient understandings of quality improvement that emerge from this study.

## Methods

This research is part of a larger ethnographic project exploring the patient involvement activities of the Collaboration for Leadership in Applied Health Research and Care for North West London (CLAHRC NWL). CLAHRC NWL uses specific quality improvement methods to help the UK National Health Service (NHS) develop and test innovative ways to improve quality of healthcare (e.g. using ‘Plan-Do-Study-Act’ cycles and sustainability models [35]), including involving patients as members of improvement teams along with 8–10 multidisciplinary frontline staff from NHS organisations [36,37].

Here we draw on 23 in-depth 60–120 minute individual interviews (10 women, 13 men) with ‘patient participants’– service users or members of the public involved in CLAHRC NWL quality improvement projects – and intensive ethnographic work including planned observation (132 hours) of official patient and public involvement activities from 2009 to 2013. These included: monthly meetings of healthcare professionals and patients working together on healthcare improvement projects (e.g. to try to improve particular health services); steering groups where patients were consulted about commissioning CLAHRC NWL improvement projects, or discussed CLAHRC NWL programme strategy; and CLAHRC NWL-funded training for patient participants to help them become “effective” patient representatives). AR conducted additional opportunistic observation regularly over the four-year project at other CLAHRC NWL events where patient involvement was not the main aim but where patients (including 20 interviewees) were present. These included CLAHRC NWL conferences to support healthcare teams’ learning about improvement methods (including patient involvement), and meetings to discuss the developments or future funding of the CLAHRC NWL programme. Both NHS and London School of Hygiene and Tropical Medicine Research Ethics Committees approved the study and we obtained written consent from interviewees.

Interviews covered general experiences of participating in healthcare improvement. We asked interviewees about quality improvement, e.g. how they defined their role in improving quality, about their motivations to get involved, and factors affecting their participation over time. To preserve anonymity, we omit or alter identifying participant details here, including details about their projects. All interviews were audio recorded and transcribed verbatim. Observations were recorded in field notes. We observed all interviewees during fieldwork. Interviewees were recruited through our participation in the field (i.e. we approached participants at observed events and invited them to be interviewed) and via snowballing: patient participants and healthcare professionals interviewed were asked to suggest contacts to approach for recruitment of subsequent interviews. Interviewees were typical of the patient participants we observed during fieldwork (formally or informally we talked to all of them during the intensive 4-year ethnographic work) in that almost all were white, educated, and had professional backgrounds. Their approximate mean age was 65 years (also typical). There was little evidence of patient participants coming from so-called ‘hard-to-reach’ groups. Through observation of patient involvement activities we examined how patient participants used their understandings of quality improvement when trying to shape healthcare services in collaboration with healthcare professionals. Following Emerson et al. [38] our observations focused on processes and practical aspects of participation in quality improvement: What actually happens when patients participate? What types of actions do patients take to try to shape quality improvement? Both authors are academics working in a Higher Education Institution, and thus started fieldwork with an outsider perspective. AR’s intensive fieldwork over the four-year period enabled her to immerse herself in the health services culture and gave her an experientially-rooted insight into quality improvement and patient involvement initiatives. The familiarity and insider perspective that AR, who conducted all the interviews and most of the field observations, gained over this four-year period was compensated with CM distance from the field. We adopted a grounded theory approach in that we used an iterative and reflexive process of collecting and analysing data and re-evaluated and questioned insights developed, with leads emerging from the analysis informing new research questions, data collection and further analysis. We analysed interviews and field notes using iterative thematic analysis [39] to identify key themes in interviewees’ experiences of participating in quality improvement work. Our coding frame reflected our a priori interest in patient definitions of quality improvement (including personal priorities for improvement, practical contributions to improvement work and views on quality improvement methodologies), and was also developed inductively from the entire data set. For instance, codes included: ‘connecting’, ‘integrating efforts’, ‘educating patients’, which spoke about the actions patient participants developed to try to influence quality improvement. During repeated rounds of coding, re-coding, and “memo-writing” [40], we made frequent comparisons across codes and the interview and field note data. Participants’ accounts were characterised by a recurrent shift in focus from healthcare systems to the patient individual level and were permeated by accountability claims and moral tones which emphasised the role of the individual patient in quality healthcare. In light of this we adopted a complementary discursive analytical approach [41] to pay closer attention to the discursive nature of interviewees’ accounts and their performative function. That is, we focused on what participants were doing when deploying their accounts: blaming, disclaiming, justifying, excusing and so on. This discursive approach illuminated how participants’ accounts and their own participatory actions to try to influence quality mobilised particular notions of quality improvement and how these notions functioned to attribute responsibility and blame for problems of quality.

We draw on social representations theory [42] to examine how patients construct notions of quality improvement. Underpinned by a social constructivist and discourse-oriented epistemology [43], this theory conceptualises social knowledge as developed through communicative interaction and social practices with others [42] and as simultaneously being shaped by and reflecting the socio-cultural and institutional contexts in which it emerges [44]. The theory helps us capture the plural and hybrid nature of social knowledge [45] and its grounding in individuals’ positions in diverse environments and relationships. The individual uses multiple and, at times, conflicting forms of thinking, practices and meanings to make sense of and organize his or her experience around the same object (in our case ‘quality improvement’) [46-48].

## Results

When patient participants tried to influence quality improvement or discussed different dimensions of quality improvement their accounts and actions frequently started with talk about improvement as dependent on collective action (e.g. multidisciplinary healthcare professionals and the public), but usually shifted away from that and towards a discourse with emphasis on the role of individual patients and the pursuit of self-improvement via promotion of behavioural change. The two discourses i.e. ideas about collectivity versus those about the individual coexisted within individual participants’ accounts, with the latter being more frequently and recurrently drawn upon to attribute responsibility for problems of quality. The two discourses are analytically discernible, however, and we present them here in turn. We discuss the second discourse in greatest detail here i.e. attributions of responsibility for problems of quality, first because this reflects its more frequent and recurring position in the data, and second because of its greater internal complexity and utility for illuminating questions of patient understandings of ‘quality improvement’ and ‘quality’.

### Collectivising quality

Quality improvement for participants was to be achieved and sustained through collaborative relationships between and amongst healthcare professionals and patients. Participants constructed quality improvement as involving interdependencies and where multidisciplinarity and non-hierarchical co-operative relationships were key to ensuring speed and spread of implementation. Their accounts reflected the “whole system” approach and collaborative ethos of the quality improvement culture [49].

At observed quality improvement team meetings and interviews, patients functioned as a “technology of persuasion” [50] for collective action in quality improvement, encouraging and facilitating cooperation between different healthcare professionals. Patients often called for more inter-organisational collaboration (e.g. between primary and secondary care) to spread the improvement interventions they helped develop to other areas more rapidly. They sometimes acted outside the official remit of their participant role to try to facilitate a collectivist approach to improving quality (Quote 1). Patients facilitated links between different healthcare professionals, patients and community groups, establishing connections at different levels (e.g. from local to national). They also worked to increase patient demand for quality healthcare to help speed up and sustain quality (e.g. helping publicise the work of the quality improvement interventions they helped develop). For instance, participant H (Quote 1) below synchronized actions between different service improvement teams he participated in (e.g. improvement of diabetes care pathways), taking ideas and information from one to the other and looking for commonalities between them to avoid duplicating efforts. He went to community healthcare services and patient groups to raise awareness of the improved hospital service hoping to help its implementation become institutionalised by increasing public demand and referrals.

*I sit on that panel [Integrated Care Pathway] and we are currently just doing all the pathways now for all of these people to ensure that they are actually getting the best treatment and best services that is available within the NHS. And of course included into that now is the [quality improvement] project. So through this I’ve now got them [the panel] to agree that we will integrate a lot of this stuff into the Integrated Care Pathway [which the panel is designing]. […] To bring in some of this information [from the quality improvement project] into their pathways. [….] I suggested taking it [the quality improvement project] to the roadshows [health promotion community event] where we would pick up more people […] and that way we can progress it […] Because I sit on that one [panel] I can take this [quality improvement project] information to that and give it out at the meetings and say look this is what this is doing at the moment. Because […] some of these things will be well and truly integrated together. […] Why reinvent the wheel when it’s not necessary […] So using that will help that*.

**Quote 1, Participant H**

At observed quality improvement meetings, participant H often asked clinicians and community nurses to collaborate to ensure the sustainability of the intervention over time (e.g. ensuring referrals from community services so that patients could benefit from the newly implemented hospital service). Participant C (below) tried to encourage a collective endeavour between healthcare professionals, patients, pharmacists and national patient charities to implement and spread a patient self-care management tool she helped develop (Quote 2).

*I’m very friendly with the pharmacist […] when I showed him the first draft [of the tool], he said Birmingham [their head office] has got to hear about this […] [The National patient charity] would probably buy into it. I’ve talked to [the charity] and they like it very much […] LINk [Local Involvement Network] are very interested in it and our doctor’s surgery […] they [the surgery] are extremely interested in it and, of course, the nursing homes are. […] he [clinician from improvement team] kept on saying, well you can’t let this one have it [the tool] and you can’t tell this one [about the tool] because, until we have all the rights sorted out […] I keep on telling them [doctors] about the [tool] and I keep on saying to them, ask them [patients] and let us know how many people actually come back to you with the [tool] […] We don’t just want it in London, we want it all over the place. […] My sister lives in [city in Scotland], […] so she’s hoping that somehow it will go north of the border.*

**Quote 2, Participant C**

Participant C tried to engage all these different stakeholders ‘unofficially’ despite the fact that the launch of the tool had been postponed because of copyright concerns. Other participants tried to engage powerful decision makers, for instance by using their participation in commissioning and parliamentary groups to raise the profile of their service improvement projects with the hope this would help the improved services expand to other settings.

Ideas about collectivism permeated participants’ accounts of their own contributions to quality improvement: they were reluctant to attribute ‘success’ to individuals, emphasising the role of the team and the synergistic efforts of team members (Quote 3). They often spoke using plurals, referring to “us” and “ours” expressing interdependence between quality improvement team members (patients and multidisciplinary healthcare professionals). Participant A below (Quote 3) uses a subtle exaggeration (‘I’ve always said”) and rejects the potential interpretation of having self-interest in the statement that they’ve won prizes for the best patient involvement work (i.e. engages in so-called ‘stake inoculation’ [51]) (‘Not that I’m saying it’s all me’). The combination has the rhetorical effect of fending off the implication that he seeks credit for his contributions to quality improvement*.*

*We’ve won the prizes for the best PPI. Not that I’m saying it’s all me but it’s a, it’s a team effort […] we’re a team and I’ve always said, there’s no ‘I’ in team. So although we win the prize for the best PPI, which would indicate that perhaps it was because of my involvement, it isn’t. I’m only part of the whole picture. I’m part of the team. […] I’m not worried about getting recognition for anything […].*

**Quote 3, Participant A**

Participant A uses talk about collectivism in his account (“we”, “it’s a team effort”, “there’s no ‘I’ in team”), but also refers to himself several times, which has the effect of hinting that his individual contribution was important to the success of PPI. As well as extolling ideas about collectivism, then, he perhaps uses the rhetoric of collectivism to take credit for his individual work in an indirect, culturally-acceptable way. In containing ideas about individual competence and responsibility, the quote illustrates how two competing discourses – ‘collectivising quality’ versus ‘self-governing health’ (explained in more detail below) – can coexist in a single narrative.

### Self-governing health

Patient participants’ accounts of how quality healthcare and quality improvements should be planned and organised collaboratively through interdependent actors, and their actions to try to facilitate this, were, somewhat paradoxically, recounted with recurrent reference to a neoliberal narrative emphasising individual patient responsibility and self-discipline. Here quality improvement was reframed as a form of governmentality of individual citizens; a call for the rational governance of personal health (what Fox et al. call a “project of self-governance” ([52]: 1307)). This narrative acted as a way of producing the type of patient worthy of quality care. It worked to move attention away from quality as dependent on or located in the effective performance of services, and towards a focus on needing to work upon individual patients to inculcate them with improved behaviours and attitudes. This neoliberal narrative operated through “practices of the self” [25: 71]. That is, it was manifested at the level of the person through practices designed to act upon the patient self (e.g. self-improvement). Patient participants focused to a large extent on promoting *being* the type of patients required to achieve quality care by creating new goals and activities for their quality improvement projects: they tried to improve care at the individual level (promoting self-care management) and increase patient responsibility for their own care.

Improvements in quality care required a ‘project of self-governance’ and the individualisation of responsibility for health. It involved autonomous patients meeting certain responsibilities in order that the healthcare system might function. Across different participants’ accounts was a recurrent emphasis on patients needing to be responsible (Quote 4) and generally the need for patients themselves to improve. Patient participants appeared to be adopting the same or similar neoliberal discourses of self-management and ‘expert-patient’ approaches widely used in contemporary service provision. Their arguments about quality improvement were tied up with evaluative accounts about patients’ subjectivity, apportioning blame to patients who lacked appropriate psychological attributes (e.g. motivation, willingness to perform ‘appropriate’ behaviour) to manage their own care (Quote 4). In these discourses, rights to quality healthcare were made contingent upon individual citizens meeting their self-care responsibilities.

*The thing I’m on at the moment is to try and get people to take responsibility for their own health […] if you have a look at the state the country’s in at the moment with obesity what we’re talking about is a very large bill sooner or later down the line […] we need to educate people what they’re actually doing to themselves by eating this rubbish […] and we’re still trying to educate the public on how important their voice is […] the government has made provision for us to have the voice, why don’t we use it? […] for patients to sit feeling sorry for themselves or just playing on it and getting their wives to do all the donkey work you know isn’t on. […] We need to make people aware that what they’re doing to themselves. […]There’s no such word as ‘can’t’. Take responsibility and say, ‘I’m not going to do it’, or, ‘I am going to do it’, not: ‘I can’t’. You can do anything you want. If the mind can conceive it, the mind can achieve it. So this is what should rule our lives, is the capability of our brains. And with the right education, we could possibly achieve.*

**Quote 4 Participant A**

Interviewees used a narrative of ‘deficit’ that involved attributing disease (and in some cases its economic costs) to a patient’s attributes and personality (Quote 4, Quote 5). At times, they produced caricatures of patients through the use of ‘extreme case formulation’ [53] (e.g. patients “feeling sorry for themselves” and wives doing all the “donkey work”) to support their claims that patients needed to be self-disciplined, Quote 4) (see also Quote 6). Patients (their psychological attributes and behaviour, e.g. passivity, ignorance, recklessness) were constructed as the problem for quality improvement (Quote 5).

Underpinning participants’ narratives and actions with respect to ‘rectifying’ patients was the widespread Western cultural ideal of “self-contained individualism” ([54]: 769); that is, the idea that people act as autonomous self-contained and self-determining rational actors and that they do this independently from their socio-cultural and political surroundings (“If the mind can conceive it, the mind can achieve it.”, Quote 4). The self-contained and self-controlling patient makes the right choices and deploys the right behaviours to take responsibility for his or her personal health [55].

*There are very, very severe issues for healthcare […] huge problems, and patients are just ignoring the reality. There are also issues with the misuse of antibiotics […] and we’re going to face a problem where we have more and more drug resistance. And pretty soon we’ll have all kinds of infections that we won’t be able to treat. And people seem to ignore that. […] There is a lot of work going on, and pharmacists, microbiologists, doctors, are spending a lot of time trying to improve the situation. Meanwhile patients couldn’t care less, and patients will buy antibiotics on the internet, they will buy antibiotics over the counter, they will… not finish their course, behave very badly, basically […] This is a big cultural problem when the patients are passive. This is something we need to change as soon as we can, for the patients, be more involved in self-care and self-management. […] Changing patient behaviour is very important.*

**Quote 5, Participant B**

Educating patients and motivating them to take control of their own health were seen as the solution to healthcare improvement – i.e. as a way to manage patients and bring about individual change to improve their health (Quote 5, Quote 6). For instance participant B’s discussion of the problem of healthcare moved on to reformulate it into a matter of “reducing the bill”, blaming patients for public expenditure and asserting that is “vital that patients start understanding the economics of healthcare”.

Participants’ patient and public involvement actions also reflected this desire to improve patients and to inculcate them with a neoliberal ideal of patient citizenship [27]. Participants embraced patient education as one of their unofficial duties within their healthcare service improvement projects (i.e. projects focused on improving care pathways or specific healthcare services). They went out into the community to raise awareness about the importance of active self-management of chronic conditions, about appropriate access to services and about adopting healthy lifestyles. Participant C (below) changed the initial focus of the service improvement project she participated in. The project’s original aim was to improve service providers’ practices, and through her involvement she shifted it into the development of an intervention to improve patients’ self-care practices and to support patients in taking ownership of their condition.

*Empower them [patients] to help themselves, that was my whole idea when I got involved in this [improvement project] because we’ve got to get over nose wipe and bottom wipe and whatever you want with patients, we have got to tell them: ‘now is the time to help yourself’.*

**Quote 6 Participant C**

Underscoring participant D’s account below is a commonly shared portrayal of patients as autonomous individuals and rational decision-makers making choices about engaging in appropriate health-related practices or not (Quote 7).

*You can’t make them* [patients] *understand it if they don’t want to, well they’ll understand but whether they want to do anything with the information they’ve got is entirely up to them. And that’s their personal choice, but for me, it is enough to be able to make sure that people have been given the right information […] And if people can understand that part of it, then they are themselves going to be improved in their healthcare […] Once people are properly made aware and then educated, then they have a choice as to whether they want to do anything about it, or whether they want to totally ignore all that. If they do, that’s theirs… but it’s, at the end of the day, also their own responsibility. […] you’ve got to instil that responsibility into the people themselves and so this comes through the education.*

**Quote 7, Participant D**

Appeals to the public to become actively involved in healthcare improvement emerged in conjunction with participants’ attempts to display their own credentials as responsible citizens who invest their personal effort and act in the public interest (i.e. the public right for quality healthcare).

Neoliberal values also operated at the level of participants’ presentation of themselves in the course of the interview. Participants invoked two distinct agentic positions; one as responsible for effectively managing their own health, and other as actively committed to patient and public involvement work (Quote 8). Both positionings cast participants as self-governing and disciplined, making personal efforts to fulfil their social duty, in contrast with an imagined mass of irresponsible patients (Quote 11). For participant D above, “it is enough” to ensure (through his patient and public involvement practices) that he provides patients with the right education. Yet it is then their “personal choice” to fulfil their responsibility “toward yourself as well as your doctor”. Participant D distinguished his own responsible actions (e.g. commitment to educating the public) from the positions adopted by a generalised patient population, which he also criticised for not engaging with public involvement initiatives. Participants treated disengagement from patient and public involvement as a marker of poor civic responsibility.

*I was thinking of a way of getting through that [illness] and dealing with things and, you know, and not being part of the problem, I wanted to be part of the solution, I just wanted to be a better patient, I wanted to get out there, I wanted to go back to work [doing PPI] […] not from a personal gratification but because I think that’s the way, if we all, if we all play a part in this, even if it’s a small part, it’ll lead to something better. Well, that’s my hope.*

**Quote 8, Participant E**

*The hardest thing is finding people that are actually motivated and proactive and people that really genuinely want to do it [patient and public involvement]. So you’ve got to be committed and that’s the hardest thing is finding people because they’re giving up their own time […] What really drives me is I don’t want to vegetate and I don’t want to sit at home watching Jeremy Kyle every day. […] I welcomed it [patient and public involvement] with open arms once the government said that we could, we were given a voice. ‘Hey guys, patients can now speak up for themselves’. So I jumped on the bandwagon straight away. […] People do a lot of complaining but they’re not prepared to do anything about it. […] 80% of the population are watchers, they’re sheep. Only 20% of the population ever become successful, those are the doers. […] No, I, I think what we need to do is to change society’s attitudes.*

**Quote 9, Participant A**

Participant A above contrasts the apathy of the general public with his commitment and drive to improve healthcare (Quote 9). He caricatures his potential patient self as sitting at home “vegetat[ing]” and “watching Jeremy Kyle”. He uses this ‘extreme case formulation’ [53] of the passive patient to create the discursive space to support his subsequent claim that patients ought to change and become “doers”. Interviewees made frequent references to the personal effort they were investing in public involvement activities, with some constructing it as a sacrifice (Quote 10, Quote 11). One participant, for instance, talked about how he had to be “disciplined” to be able to fulfil his responsibilities as a “lay member" of a research ethics committee, and suggested that right to quality care was dependent upon patients fulfilling their public involvement responsibilities. Extreme examples of the way participants regulated their own involvement efforts are two participants who talked about the health and economic costs of involvement.

*I’m thinking of slowing down a bit because I’ve got myself rather lumbered with so many [PPI] meetings […] I can either do that [PPI], or start being entirely selfish and just go round travelling and doing personally, pleasing myself or getting foot massages […] It’s a decision [doing PPI]…It’s a difference in character between giving or taking […] You can exceed your parameters with the amount that you give […] I don’t mind giving, you know.*

**Quote 10, Participant F**

*I really wanted to get well but I felt that it was my social duty to get well, to get back to work if I possibly could. And so I tried everything that you could think of […] You may very well live on benefits and feel guilty about it. […] Instead of just sitting at home and moaning that they don’t have more than what they have and not get involved […] My main stumbling block [when doing PPI] is my own health condition which renders me utterly exhausted and my energy levels are tremendously impaired […] that is not something anybody can help me with. Either I can do something or I can’t do it or and then I pay the price afterwards, but I was prepared to pay the price.*

**Quote 11, Participant G**

## Discussion

Our findings illustrate the subtle ways in which neoliberal ideals circulating in the environments where patients receive care operate ‘at a distance’ [56,57] (i.e. by permeating their common sense understandings and promoting particular behaviours as ‘naturally’ better) and the processes by which its ideological assumptions about choice, responsibility and rational autonomy are manifested at the local level of individual patients. Such governance ‘at a distance’ is manifest in the way patients construct ideas of ‘quality improvement’ when participating in shaping healthcare services, and it also plays out in their own subjectivity (i.e. their perceptions and enactment of their roles as patients and as participants in quality improvement).

Typically, definitions of quality healthcare focus on the performance of healthcare systems (e.g. The Institute of Medicine [58]). Yet when discussing quality improvement or trying to influence the quality of healthcare services, participants emphasised the role of individual patients and the pursuit of self-improvement instead. Although they also adopted collectivist approaches (e.g. by trying to enhance collaboration), these co-existed with a recurrent focus on quality improvement as a “project of self-governance” [52: 1307] to improve the personal attributes and behaviours of citizens. This neoliberal governance ‘at a distance’ also permeated the way participants presented their own subjectivities as patients and as public involvement participants. They positioned themselves as agents willing to take, and choosing to take, responsibility for the management of their own health and for participating in improving care for others (i.e. fulfilling their citizenship duties). Participants portrayed themselves as governed by self-discipline and personal effort in their patient and public involvement work, and in doing so provided examples of how neoliberal appeals for self-regulation and self-determination permeate participants’ own positionings.

The juxtaposition of neoliberal discourses with collectivist approaches in participants’ accounts and actions reflects the ways in which citizens appropriate, negotiate and enact the tensions arising from different policies and agendas [23] when making sense of healthcare quality improvement. On the one hand, neoliberal policies and models of healthcare individualise responsibility for the management of health and position patients as autonomous rational actors [59] presuming they can act independently within the broader social contexts and structural conditions which shape their health and illness [60,61]. On the other, the relational approach of quality improvement is based on the idea of “collaboratives” [12] and networks of interdependent stakeholders (including patients and multidisciplinary professionals) working together to deliver high quality care [31]. The latter, collectivist, approach is at the heart of the quality improvement culture of the programme in which patients in our study participated. We have shown before how patient participants use elements of this culture as resources to make sense of their role and facilitate their involvement [49]. This paper highlights how they also partially draw upon the collectivist emphasis of the improvement programme to construct and enact notions of quality improvement, e.g. in attempting to influence quality improvement, patient participants also catalysed collective action, stimulating collaboration and interdependencies between healthcare professionals, patients and civil society, and establishing connections at different levels. Their “interconnecting tactics” [62] can potentially make quality improvement a more collective effort by engaging the networks of people and actions needed for delivering sustained improvement. Yet the underlying presumption in patient participant narratives of the self-contained person who makes rational decisions to take control of his or her health may be problematic. First, because it could discourage patients from recognising the importance of interdependencies and collaborative working between multiple stakeholders from different sectors and contexts thus limit patient participants’ roles in catalysing more collaborative forms of healthcare improvement; and second, because psychologising health, that is, reducing health problems to deficits in the individual and neglecting upstream determinants of health runs the risk of justifying inequalities in health and perpetuating the status quo [63]. Neoliberal ideals can operate at a distance by influencing patient perspectives and potentially constraining their expectations and hopes about what the state can do or should do to cater for their rights to quality healthcare. This in turn can limit the agency of patients to influence and claim their rights to quality healthcare, in other words, to exercise their ‘participatory citizenship’ in healthcare improvement [62,64].

In relation to ‘quality’ itself, our study participants’ notions of quality improvement show that whatever patients see as important components of care, they may nevertheless consider that their right to them is contingent on individual obligations to self-govern and live one’s life in a particular way. The attribute that seems to define the notion of ‘quality’ that underpins participants’ understandings about quality improvement is its moral “appeal” [Cf. Goldenberg who refers to the "persuasive appeal" of quality of care, 9]. This moral appeal speaks about citizens working towards self-improvement to be worthy of quality care. Quality care calls on a particular patient self; someone who displays the ‘correct’ attributes and behaviours (self-determination, agency, responsibility, self-control) to manage personal health and to contribute towards improving healthcare for others through public involvement work.

The move towards neoliberal consumerist models could undermine the rise of ”activist citizen” [65]: patients both empowered to act as claimants of rights to quality care and empowered to demand the state fulfil its duties of care. Our study shows how neoliberal ideals were taken up in discourses about quality improvement in a way that removed attention from the state and healthcare providers as accountable to patients’ rights to quality care, with patients focusing instead on the need for citizens (including themselves) to work on improving themselves. The patient self was largely constructed as the subject of obligations rather than rights.

Our findings are derived from a group of patients participating in a specific quality improvement programme in one city in the UK. While we do not claim that their views represent that of all the patients across the country, our research provides insights into the way patients who are active in shaping healthcare services through public involvement initiatives construct notions of quality improvement and highlights the role social contexts may play in mediating patients’ perspectives and actions. It demonstrates the need to account for patients’ plural understandings of quality improvement in the context of the diverse health policies and cultural ideals circulating in the environments where patients receive care.

Participants in our study were typical of the patients involved in patient and public involvement activities [66] in that almost all were educated, seasoned participants and had professional backgrounds. The homogeneous nature of patient participants—and the absence of representation from so-called ‘hard-to-reach’ or underserved groups — is widely recognised in the patient involvement literature [66,67]. It is not possible to know whether patients without previous participatory experiences, or from other backgrounds, would have had similar views about quality improvement. Nevertheless, patients involved in shaping healthcare are an important group and offer a key entry point to understanding ‘quality’ and ‘quality improvement’ from a patient perspective, particularly as these questions have largely remained unexplored in any patient group. Our findings will help inform further investigation of these issues from the perspective of other less-active and non-involved patients. The similarities in social background of our study participants might be one reason for the convergence of viewpoints in our data.

## Conclusions

Neoliberal rationalities can work to produce the “common-sense” understanding [68] that there are no “rights [to quality healthcare] without responsibilities” [65,69] (i.e. the responsibility to enact idealised models of patient citizenship demanded by neoliberal health governance [27]). As our study shows, such ‘common sense’ was reflected in the way patients perceived and shaped ideas about ‘quality’. It also played out in their subjectivity in terms of the way they governed and regulated their own involvement efforts in quality improvement. Without attention to how patient perspectives of quality improvement and the notions of ‘quality’ that these illuminate are influenced ‘at a distance’ by neoliberal rationalities that may mask state duties of care, public involvement interventions and policies risk limiting how patients can shape quality improvement to respond to their own needs and preferences. When including patient voices in measuring and defining ‘quality’, governments and public health practitioners need to be aware of how neoliberal rationalities at the heart of the policy and services context could prevent patients from seeing themselves as subjects of rights to quality care and as actors “with ‘the right to claim rights” ([65]:371) to quality care. Any efforts to improve patient and public involvement should also focus on supporting members of the public to engage critically with how neoliberal rationalities can undermine their agency to act as claimants of rights to receive quality care. Helping patients to develop this “critical consciousness” [70,71] is an important first step towards their empowerment to participate [72] and is crucial to permit the democratic potential of patient and public involvement initiatives -- as well as any resulting improvements -- to be realised.
